# Suspected Lynch syndrome associated *MSH6* variants: A functional assay to determine their pathogenicity

**DOI:** 10.1371/journal.pgen.1006765

**Published:** 2017-05-22

**Authors:** Hellen Houlleberghs, Anne Goverde, Jarnick Lusseveld, Marleen Dekker, Marco J. Bruno, Fred H. Menko, Arjen R. Mensenkamp, Manon C. W. Spaander, Anja Wagner, Robert M. W. Hofstra, Hein te Riele

**Affiliations:** 1 Division of Biological Stress Response, The Netherlands Cancer Institute, Amsterdam, The Netherlands; 2 Department of Clinical Genetics, Erasmus University Medical Center, Rotterdam, The Netherlands; 3 Department of Gastroenterology and Hepatology, Erasmus University Medical Center, Rotterdam, The Netherlands; 4 Family Cancer Clinic, The Netherlands Cancer Institute, Amsterdam, The Netherlands; 5 Department of Human Genetics, Radboud University Medical Center, Nijmegen, The Netherlands; Cleveland Clinic Genomic Medicine Institute, UNITED STATES

## Abstract

Lynch syndrome (LS) is a hereditary cancer predisposition caused by inactivating mutations in DNA mismatch repair (MMR) genes. Mutations in the *MSH6* DNA MMR gene account for approximately 18% of LS cases. Many LS-associated sequence variants are nonsense and frameshift mutations that clearly abrogate MMR activity. However, missense mutations whose functional implications are unclear are also frequently seen in suspected-LS patients. To conclusively diagnose LS and enroll patients in appropriate surveillance programs to reduce morbidity as well as mortality, the functional consequences of these variants of uncertain clinical significance (VUS) must be defined. We present an oligonucleotide-directed mutagenesis screen for the identification of pathogenic *MSH6* VUS. In the screen, the *MSH6* variant of interest is introduced into mouse embryonic stem cells by site-directed mutagenesis. Subsequent selection for MMR-deficient cells using the DNA damaging agent 6-thioguanine (6TG) allows the identification of MMR abrogating VUS because solely MMR-deficient cells survive 6TG exposure. We demonstrate the efficacy of the genetic screen, investigate the phenotype of 26 *MSH6* VUS and compare our screening results to clinical data from suspected-LS patients carrying these variant alleles.

## Author summary

The colorectal and endometrial cancer predisposition Lynch syndrome (LS) is caused by an inherited heterozygous defect in one of four DNA mismatch repair (MMR) genes. Deleterious mutations (*e*.*g*., protein-deleting or -truncating) in DNA MMR genes unambiguously allow for the clinical diagnosis LS and hence enable appropriate surveillance measures to be taken to reduce cancer risk and ensure early detection of tumors. However, currently about one-third of detected MMR gene variants are subtle with less clear functional consequences: missense mutations *affecting a single amino acid* may be innocuous, hence not causing LS, or partially or fully destroy protein function. As long as uncertainty exists about their pathogenicity, such mutations are labeled *‘variants of uncertain (clinical) significance’* (VUS). VUS hamper genetic counseling and therefore the need for functional testing of VUS is widely recognized. To functionally annotate MMR gene VUS, we have developed a high content cellular assay in which the VUS is introduced in a cell culture by oligonucleotide-directed gene modification. Should the VUS be deleterious for MMR, the modified cells survive exposure to the guanine analog 6-thioguanine (6TG) and 6TG-resistant colonies appear. Should the mutation not affect MMR, no colonies appear. Here we present the adaptation and application of this protocol to the functional annotation of variants of the MMR gene *MSH6*. Implementation of our assay in clinical genetics laboratories will provide clinicians with information for proper counseling of mutation carriers and treatment of their of tumors.

## Introduction

Lynch syndrome (LS) is an autosomal-dominantly inherited predisposition to a variety of malignancies at a young age, mainly colorectal cancer (CRC) and endometrial cancer (EC) [1]. It is caused by inactivating germ-line mutations in the DNA mismatch repair (MMR) genes *MLH1*, *MSH2*, *MSH6* or *PMS2*, or a deletion in the 3’ region of the *EPCAM* gene that affects *MSH2* expression [2–6].

The DNA MMR system is essential for the fidelity of DNA replication. Its primary function is the correction of base-base mismatches and insertion-deletion loops that may arise during DNA replication. Base-base mismatches are recognized by the MSH2-MSH6 heterodimer while MSH2-MSH3 detects loops of unpaired bases. Following mismatch binding, the MSH heterodimers recruit another heterodimer, MLH1-PMS2, to coordinate removal and resynthesis of the error-containing strand [7–9]. A second function of the DNA MMR system is to mediate the toxicity of certain DNA damaging agents such as methylating agents and thiopurines. These DNA damaging agents create adducts in the genome that give rise to mismatches when replicated. The DNA MMR system recognizes the mismatches but will remove the incorporated nucleotide rather than the lesion itself, creating a repetitive cycle of nucleotide incorporation and deletion that ultimately leads to DNA breakage and cell death [10,11]. In the absence of MMR, cells tolerate methylation damage, but consequently show high levels of DNA damage-induced mutagenesis on top of a strongly elevated level of spontaneous mutagenesis [12].

LS patients inherit a functional and a mutant copy of one of the DNA MMR genes. For cells to become MMR-deficient and develop a mutator phenotype that accelerates carcinogenesis, somatic loss of the wild-type allele is required [13]. Microsatellite instability (MSI), *i*.*e*., length alterations of repetitive sequences like (CA)_n_ or (A)_n_, and loss of immunohistochemical staining (IHC) for MMR proteins are considered hallmarks of LS tumors. Analysis of MSI and IHC on tumor tissue can identify patients who may suffer from LS. For a definitive LS diagnosis, however, sequence analyses must reveal a pathogenic germline mutation in one of the DNA MMR genes or the 3’ region of *EPCAM* [14,15]. Many LS-associated sequence variants are nonsense and frameshift mutations that clearly truncate the protein and unambiguously abrogate MMR activity. Missense mutations that only alter a single amino acid are also frequently identified in suspected-LS patients. The functional implications of these variants are less clear. Consequently, the diagnosis of suspected-LS patients carrying missense variants is difficult in the absence of clear segregation and functional data. As long as the phenotype of these variants of uncertain significance (VUS) is unclear, non-carriers cannot safely be discharged from burdensome surveillance programs [16]. Surveillance programs have proven to significantly reduce morbidity and mortality in LS patients [1,17,18], but pose unnecessary psychological and physical stress on carriers of innocent VUS as well as pressure on preventive healthcare. Therefore, techniques that characterize MMR gene VUS and enable the identification of individuals at risk are urgently needed.

While in the past primarily *MSH2* and *MLH1* were sequenced to identify LS-causing mutations, in recent years *MSH6* has been gained fame for causing LS due to the advancement of DNA sequencing. However, *MSH6* mutation carriers can be difficult to diagnose because they may not entirely fulfill the criteria for LS diagnosis: their age at cancer onset is often later than for *MLH1* and *MSH2* mutation carriers, and their tumors occasionally stain for MSH6 and have no or low MSI [19–21]. We therefore extended the applicability of the oligonucleotide-directed mutagenesis screen we recently described for the identification of pathogenic *MSH2* variants to *MSH6* variants [22]. The genetic screen uses oligonucleotide-directed gene modification (oligo targeting) [23] to introduce variant codons into the endogenous *Msh2* gene of mouse embryonic stem cells (mESCs) and subsequently identifies pathogenic variants by selecting for cells that are resistant to the thiopurine 6-thioguanine (6TG). Here we present the applicability of this screen for the characterization of *MSH6* VUS.

## Results

### Genetic screen for the identification of pathogenic *MSH6* variants

**Fig 1:**
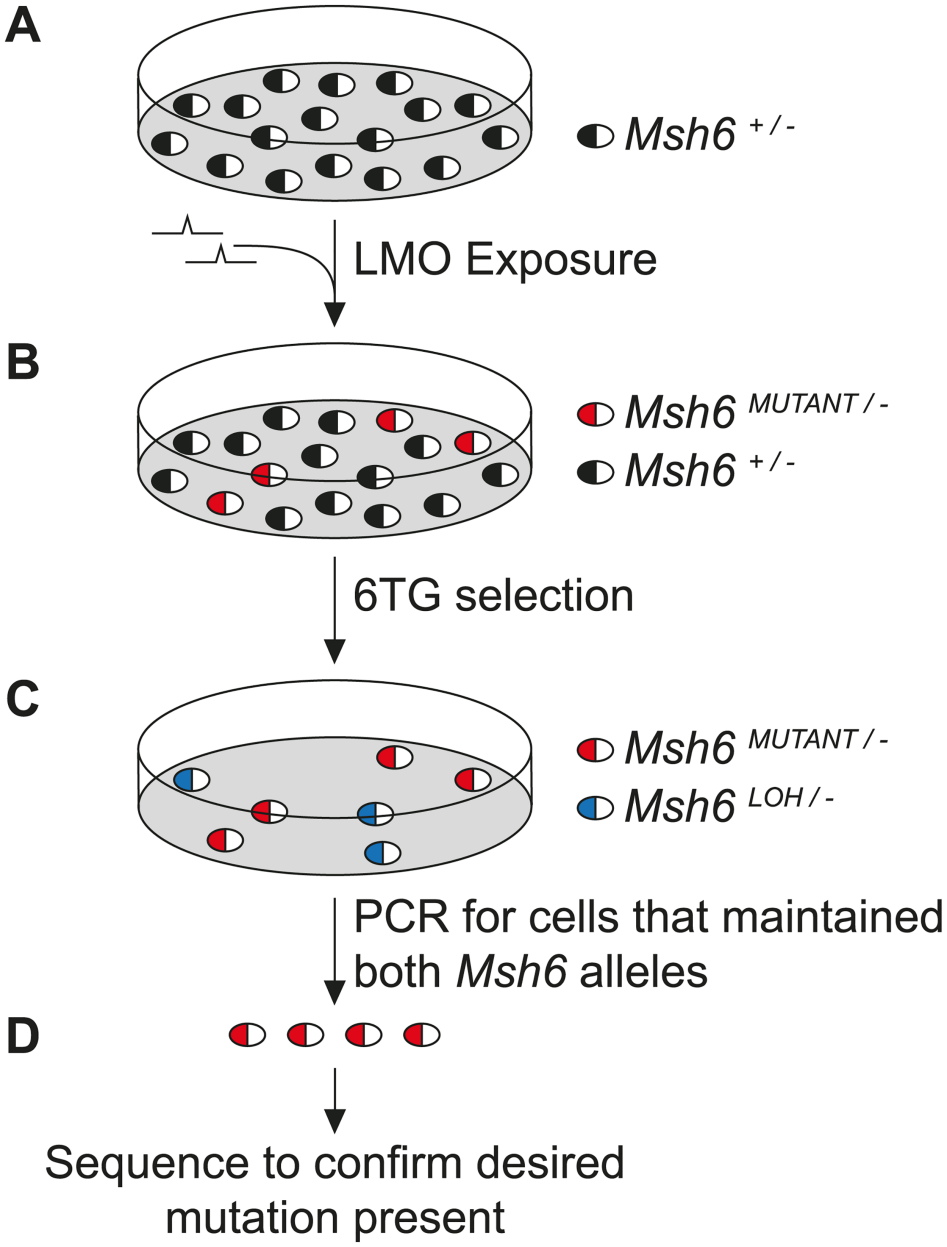
Oligonucleotide-directed mutagenesis screen for the detection of pathogenic *MSH6* variants. (A) *Msh6*^*+/-*^ mESCs were exposed to LMOs encoding the mutations of interest. LMO-exposure introduced the mutation into the endogenous *Msh6* gene in ±1 per 1000 *Msh6*^*+/-*^ mESCs. (B) To determine if the subset of cells carrying the mutation in the *Msh6*^+^ allele had lost MMR activity, the mESCs were treated with 6TG. MMR-proficient cells die in response to 6TG exposure while MMR-deficient cells are 6TG resistant. (C) Cells may also lose MMR capacity due to loss of heterozygosity (LOH) events deleting the *Msh6*^+^ allele. To exclude these cells from further investigation, a PCR was performed that detected the presence of both *Msh6* alleles. (D) 6TG-resistant LMO-exposed mESCs that maintained the *Msh6*^+^ allele were sequenced to confirm the presence of the planned mutation.

The oligonucleotide-directed mutagenesis screen takes a four step approach to the identification of pathogenic *MSH6* mutations (Fig 1): 1) site-directed mutagenesis to introduce the variant of interest into a subset of *Msh6*^*+/-*^ mESCs, 2) selection for cells that consequently lost MMR capacity, 3) PCR analysis to exclude cells that lost MMR capacity due to loss of the *Msh6*^+^ allele (loss of heterozygosity events), 4) sequence analysis to confirm the presence of the planned mutation in the MMR-deficient cells.

mESCs provide a good study model because the human and mouse MSH6 amino acid sequences share over >86% identity (S1 Fig) and mouse models can be made from these cells if VUS need to be studied *in vivo*. *Msh6*^*+/-*^ mESCs only contain one wild type *Msh6* allele (*Msh6*^+^); the other allele was disrupted by a *puromycin*-resistance gene and therefore inactivated (*Msh6*^-^) [24]. Hence introduction of a specific mutation into the one active *Msh6* allele will lead to expression of solely the variant protein and allow immediate investigation of its phenotype. To achieve this, *Msh6* was site-specifically mutated by oligo targeting, a gene modification technique that uses short single-stranded locked-nucleic-acid-modified DNA oligonucleotides (LMOs) (with either sense or antisense orientation) to substitute a single base pair at a desired location. LMO-directed base-pair substitution can be achieved at an efficiency of 10^−3^; thus, about 1 in every 1000 LMO-exposed *Msh6*^*+/-*^ mESCs will contain the desired mutation [23]. To determine whether the substitution abrogated *Msh6* activity and this subset of cells consequently lost MMR activity, LMO-exposed mESCs were treated with 6TG. The thiopurine DNA damaging agent 6TG is highly toxic to MMR-proficient but only moderately toxic to MMR-deficient cells [11]. Therefore, the appearance of colonies that survived mild 6TG selection is indicative for loss of MMR capacity. Loss of MMR capacity may arise due to the introduced mutation or due to loss of heterozygosity events that caused loss of the functional *Msh6* allele. To exclude the latter from further investigation, a PCR that detected the presence of both the disrupted and non-disrupted *Msh6* alleles was performed [24]. 6TG-resistant colonies that maintained both *Msh6* alleles were sequenced to confirm the presence of the planned mutation.

### Proof of principle

To demonstrate the ability of the oligonucleotide-directed mutagenesis screen to distinguish pathogenic *MSH6* mutations from polymorphisms, a proof of principle study was performed with *MSH6* variants G1139S and L1087R that were previously proven to be pathogenic and not pathogenic, respectively [25], as well as all classified pathogenic and not pathogenic missense variants described in the International Society for Gastrointestinal Hereditary Tumours (InSiGHT) colon cancer variant database (http://insight-group.org/). This database uses available clinical, *in vitro* and *in silico* data to categorize DNA MMR gene sequence variants according to a five-tiered classification scheme as: class 5, Pathogenic; 4, Likely pathogenic; 3, Uncertain; 2, Likely not pathogenic; and 1, Not pathogenic [26]. *Msh6*^*+/-*^ mESCs were first exposed to antisense oriented LMOs encoding the desired base-pair substitution. If subsequent 6TG selection did not reveal resistant colonies encoding the planned mutation, the screen was repeated with sense oriented LMOs.

**Fig 2:**
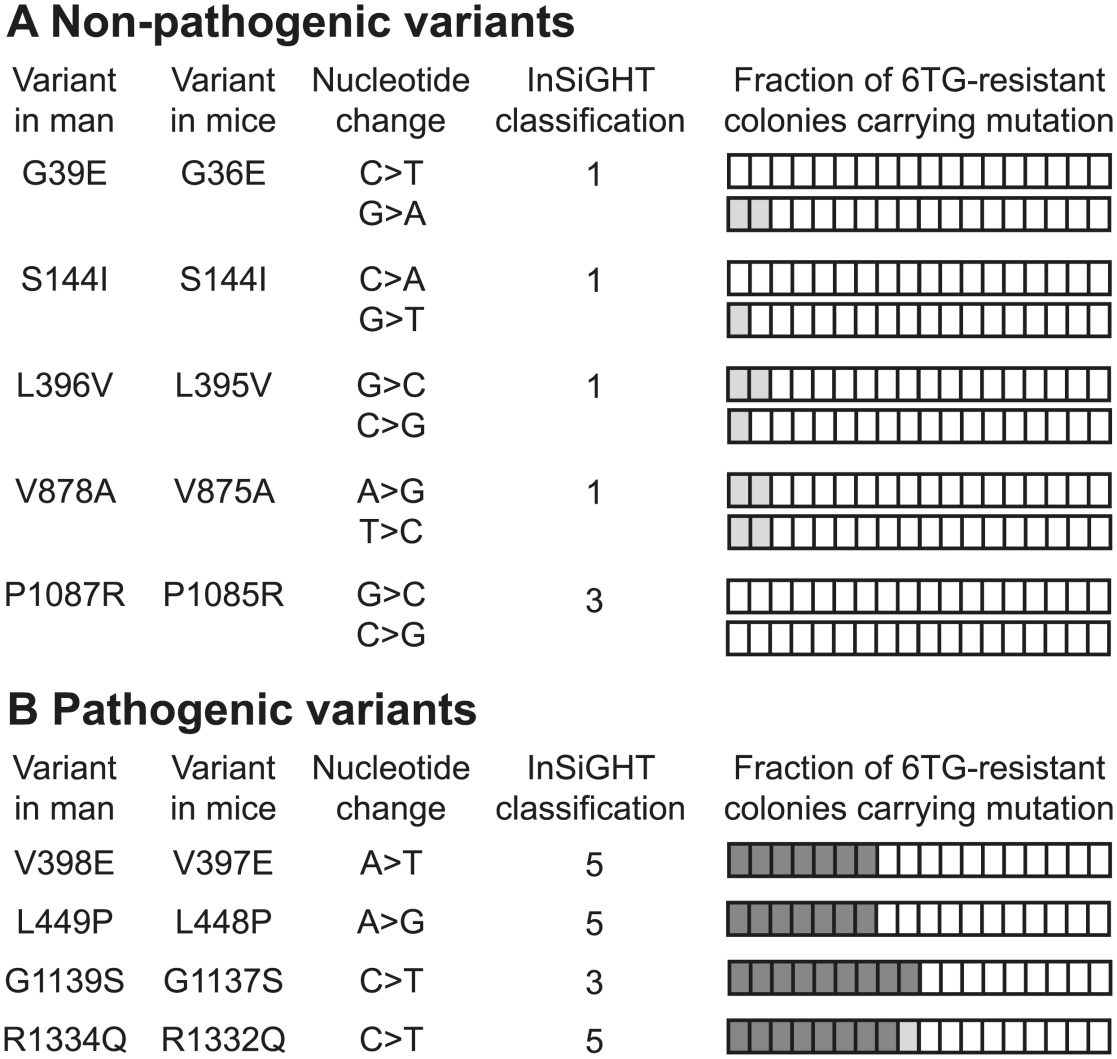
Distinguishing pathogenic *MSH6* variants from polymorphisms. (A) Five known non-pathogenic variants and (B) four pathogenic mutations tested in the proof of principle study. Variants are annotated according to their amino acid change and location in men and mice. The nucleotide change was first introduced by antisense-oriented LMOs. If no 6TG-resistant colonies encoding the mutation appeared, the screening protocol was repeated with sense-oriented LMOs (lower row where two rows are present). The fourth column presents the InSiGHT classification of each variant where 5 is pathogenic, 3 is uncertain and 1 is not pathogenic. At variance with the InSiGHT classification, a previous study demonstrated variant G1139S is pathogenic and L1087R is not pathogenic [25]. The bars in the ‘Fraction of 6TG-resistant colonies carrying mutation’ column represent the 18 6TG-resistant colonies that were investigated further. The white portions represent colonies in which the *Msh6*^+^ allele was lost by LOH; the light grey portions illustrate the fraction of background colonies that apparently survived 6TG selection but maintained the *Msh6*^+^ allele without the planned mutation; the dark grey portions represent the fractions of colonies that maintained the *Msh6*^+^ allele and encoded the mutation of interest.

LMO-mediated introduction of both, pathogenic and not pathogenic variants led to the appearance of 6TG-resistant colonies. For each LMO, we picked and analyzed 18 colonies. The vast majority of 6TG-resistant colonies obtained with LMOs encoding *polymorphisms* had lost the wild-type *Msh6* allele by loss of heterozygosity (LOH) events, as inferred from allele-specific PCR analysis. Sequencing of the few 6TG-resistant colonies that had retained both *Msh6* alleles (±6%), did not detect any mutation (Fig 2A). These background colonies apparently arose from cells that for unknown reasons survived 6TG exposure. Of the 6TG-resistant colonies that emerged following LMO-mediated introduction of *pathogenic mutations*, ±40% still contained both *Msh6* alleles. Sequence analysis detected pathogenic mutations in all but one of these 6TG-resistant colonies (Fig 2B; S2A Fig). Thus, the oligonucleotide-directed mutagenesis screen detected all 4 pathogenic mutations and not one of the 5 non-pathogenic variants, indicating it is capable of distinguishing pathogenic *MSH6* mutations from polymorphisms.

### Screening variants of uncertain significance

**Fig 3:**
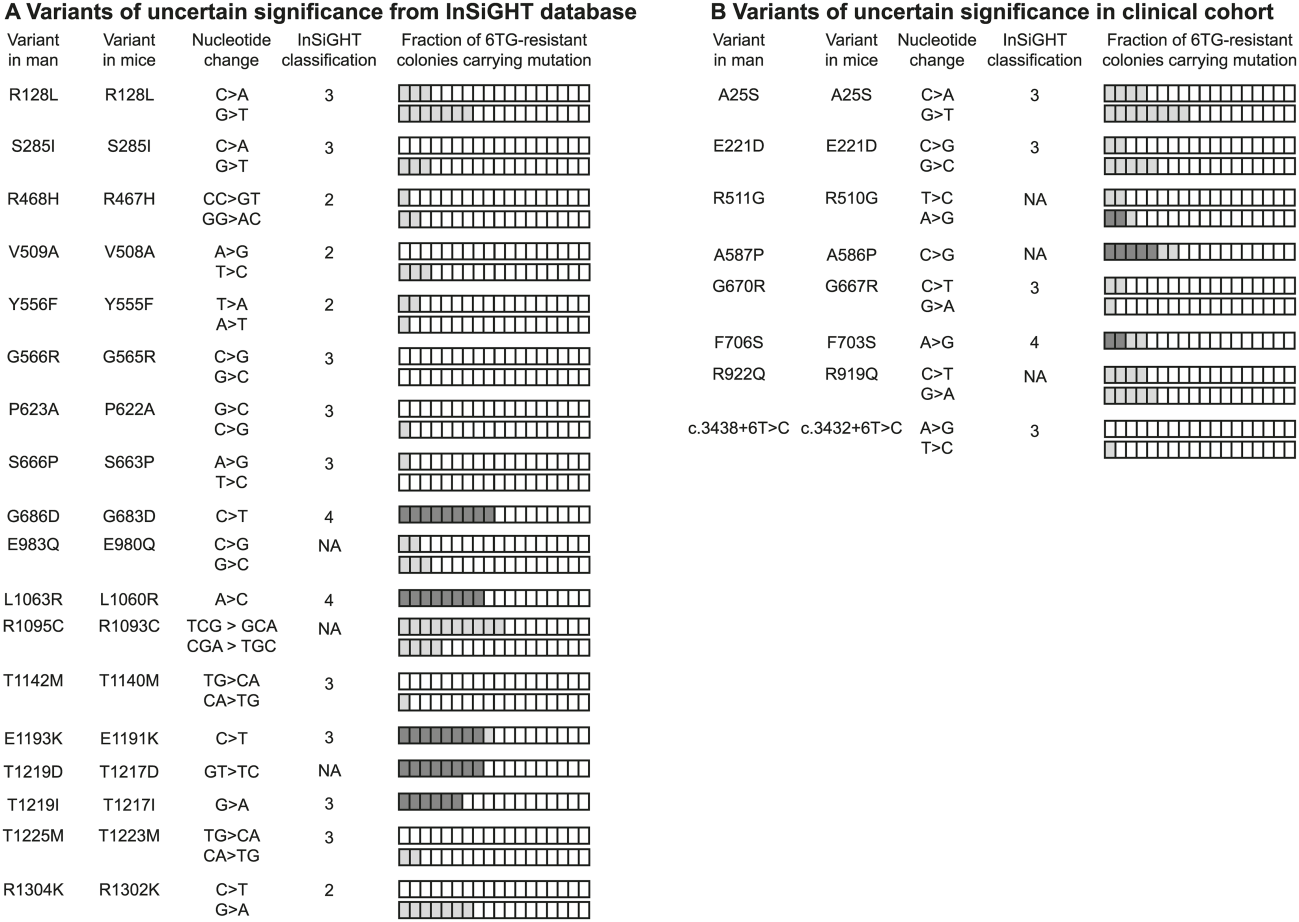
Identification of pathogenic *MSH6* VUS. The genetic screen was used to analyze (A) 18 VUS selected from literature and the InSiGHT database as well as (B) 8 VUS identified in patients from two medical centers in the Netherlands. Variants are displayed according to their amino acid number and change in men and mice. The ‘Nucleotide change’ column presents the one or two base alteration introduced by the LMOs. If antisense-oriented LMOs did not give rise to 6TG-resistant colonies encoding the mutation of interest, the screen was repeated with sense-oriented LMOs (lower row where two rows are present for the variant). The InSiGHT classification of each variant is indicated: 4, likely pathogenic; 3, uncertain; 2, likely not pathogenic; NA, not available. The bars in the ‘Fraction of 6TG-resistant colonies carrying mutation’ column represent the 18 6TG-resistant colonies that were analyzed for the presence of the planned mutation: the white segments represent LOH events; the light grey segments represent background colonies that maintained the *Msh6*^+^ allele but did not encode the planned mutation; the dark grey segments display the fractions of colonies that maintained the *Msh6*^+^ allele and encoded the mutations of interest.

We used the oligonucleotide-directed mutagenesis screen to investigate the phenotype of 18 *MSH6* VUS described in literature and the InSiGHT database as well as 8 *MSH6* VUS detected in suspected-LS patients from the Erasmus Medical Center Rotterdam and the Radboud University Medical Center Nijmegen (see S1 and S2 Tables for clinical data [27–38]; see S3 Fig for location of variants in MSH6 [39,40]). Of the 26 variants, 18 were not present in 6TG-resistant colonies and hence do not appear to affect MMR activity. Mutations R510G, A586P, G683D, F703S, L1060R, E1191K, T1217D and T1217I were identified in 6TG-resistant colonies by sequence analysis (Fig 3A and 3B; S2B Fig). Of note, variants R510G and F703S were detected in only two colonies out of five and four, respectively, that had not resulted from LOH (Fig 3B). Given the low frequency of LMO-mediated base-pair substitution, we consider the presence of a variant allele in two independent colonies indicative for pathogenicity. The MMR abrogating effect of all *Msh6* variants conferring 6TG-resistance was further characterized by Western blot analyses, MSI assays and methylation-damage-induced mutagenesis assays.

### Phenotypic assessment of identified MMR abrogating *Msh6* variants

**Fig 4:**
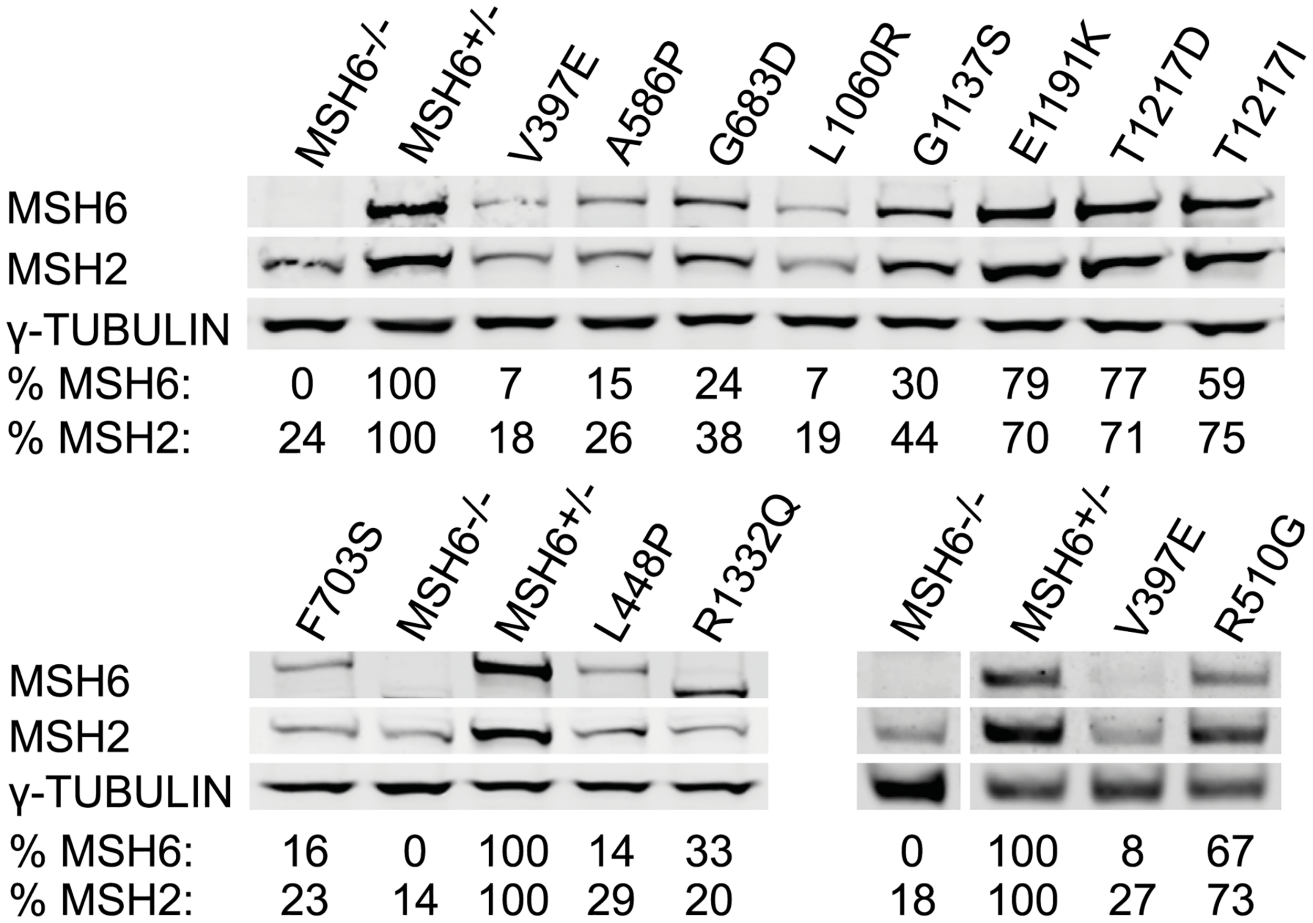
Western blot analysis of mESCs expressing *Msh6* variants. MSH6, MSH2 and γ-TUBULIN levels were analyzed in whole cell lysates. MSH6 and MSH2 levels in the variant cells lines were quantified with respect to the protein levels seen in *Msh6*^*+/-*^ mESCs.

The effect of the identified MMR abrogating mutations on MSH6 and MSH2 protein levels was evaluated by Western blot analyses (Fig 4). MSH6 and MSH2 form a heterodimer; consequently, a drop in MSH6 levels is often associated with a slight decrease in MSH2 protein stability. Protein levels were quantified with respect to *Msh6*^*+/-*^ mESCs, which maintain a functional MMR system with about two-third of the MSH6 level observed in *Msh6*^*+/+*^ mESCs [25]. Known pathogenic mutations V397E, L448P, G1137S and R1332Q reduced MSH6 levels to 7–33% of that seen in *Msh6*^*+/-*^ mESCs. The R1332Q mutation is located in the splice donor site of exon 9 which may explain the appearance of a shorter protein. The drop in MSH6 levels seen for the known pathogenic mutations was mirrored by variants A586P, G683D, F703S and L1060R that reduced protein levels to 7–24%. Variants R510G, E1191K, T1217D and T1217I maintained relatively high MSH6 levels of 59–79%.

**Fig 5:**
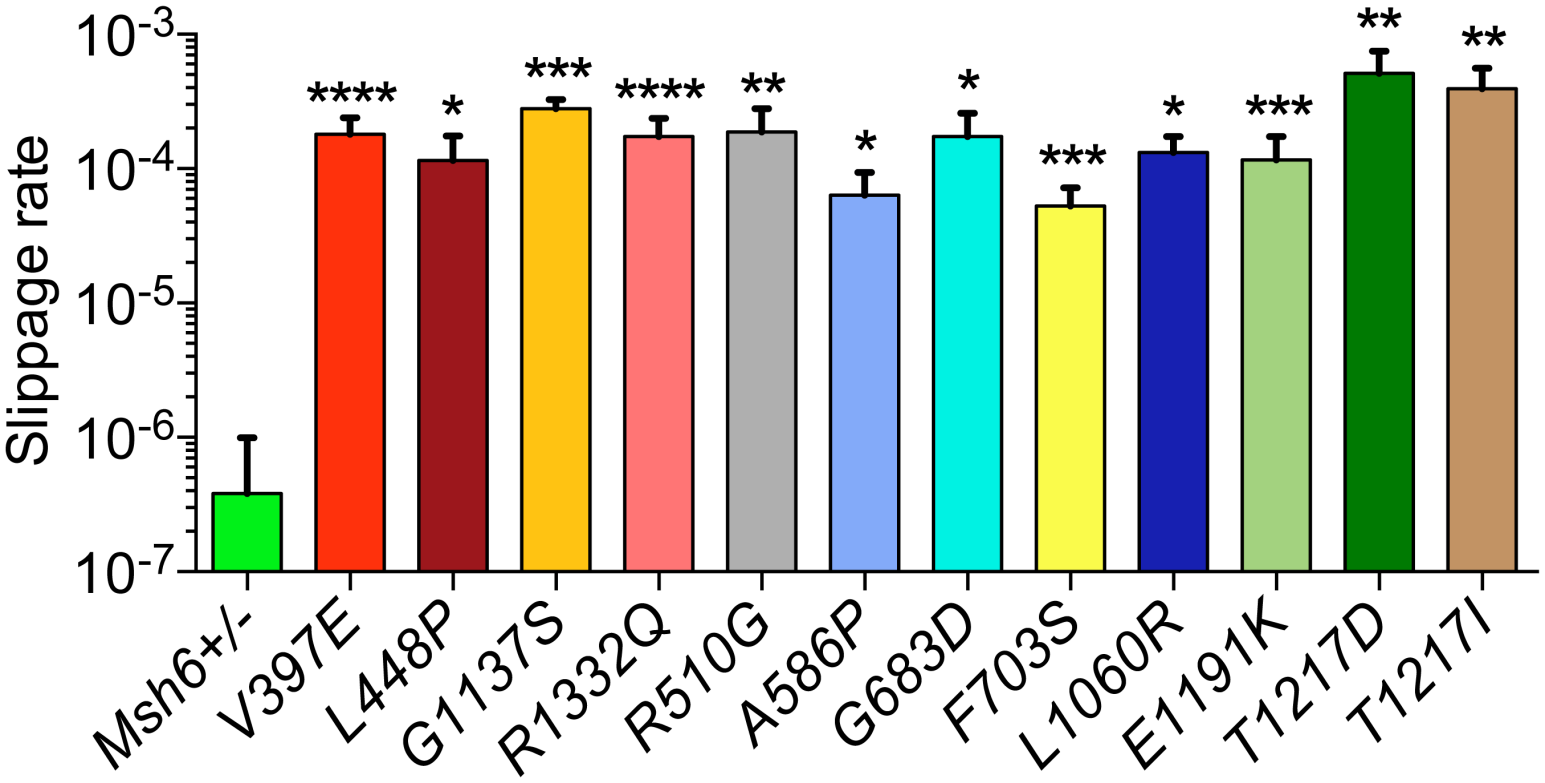
MSI analysis of mESCs expressing *Msh6* variants. To quantify the level of MSI, a (G)_10_-*neo* slippage reporter was introduced into variant mESCs. Spontaneous DNA polymerase slippage events on the (G)_10_ repeat that are not corrected can bring the *neo* in frame, rendering cells Geneticin-resistant. Slippage rates (the emergence of a Geneticin-resistant cell per cell division) of VUS expressing cells are compared to the MMR-proficient *Msh6*^*+/-*^ cell line and MMR-deficient *Msh6* ^*V397E/-*^, *Msh6* ^*L448P/-*^, *Msh6* ^*G1137S/-*^, and *Msh6* ^*R1332Q/-*^ pathogenic controls. Statistical differences were calculated using one-tailed, unpaired t-test with Welch’s correction. Asterisks indicate values significantly higher than those of the MMR-proficient *Msh6*^*+/-*^ control: *P<0.05; **P<0.01; ***P<0.001; ****P<0.0001.

MSI in *MSH6* mutation carriers is largely restricted to mononucleotide markers [41]. To investigate the effect of the detected *Msh6* variants on MSI we used a (G)_10_-*neo* slippage reporter. The neomycin resistance gene (*neo*) in this reporter is rendered out of frame by a preceding (G)_10_ repeat. When DNA polymerase slippage errors at the (G)_10_ repeat such as the deletion of one G or insertion of two Gs remain unnoticed, the *neo* becomes in frame and generates Geneticin-resistant cells. Hence the number of Geneticin-resistant colonies is indicative of the frequency of *neo*-restoring slippage events and the MMR capacity of the cells [42]. The slippage rates, *i*.*e*., the chance of a slippage event occurring during one cell division, in 6TG-resisant *Msh6* VUS expressing mESCs ranged from 5.3x10^-5^ to 5.1x10^-4^; which is around the average rate of 1.9x10^-4^ observed for the known pathogenic mutations and 140 to 1340-fold higher than the slippage rate of 3.8x10^-7^ seen for *Msh6*^*+/-*^ MMR-proficient mESCs (Fig 5).

**Fig 6:**
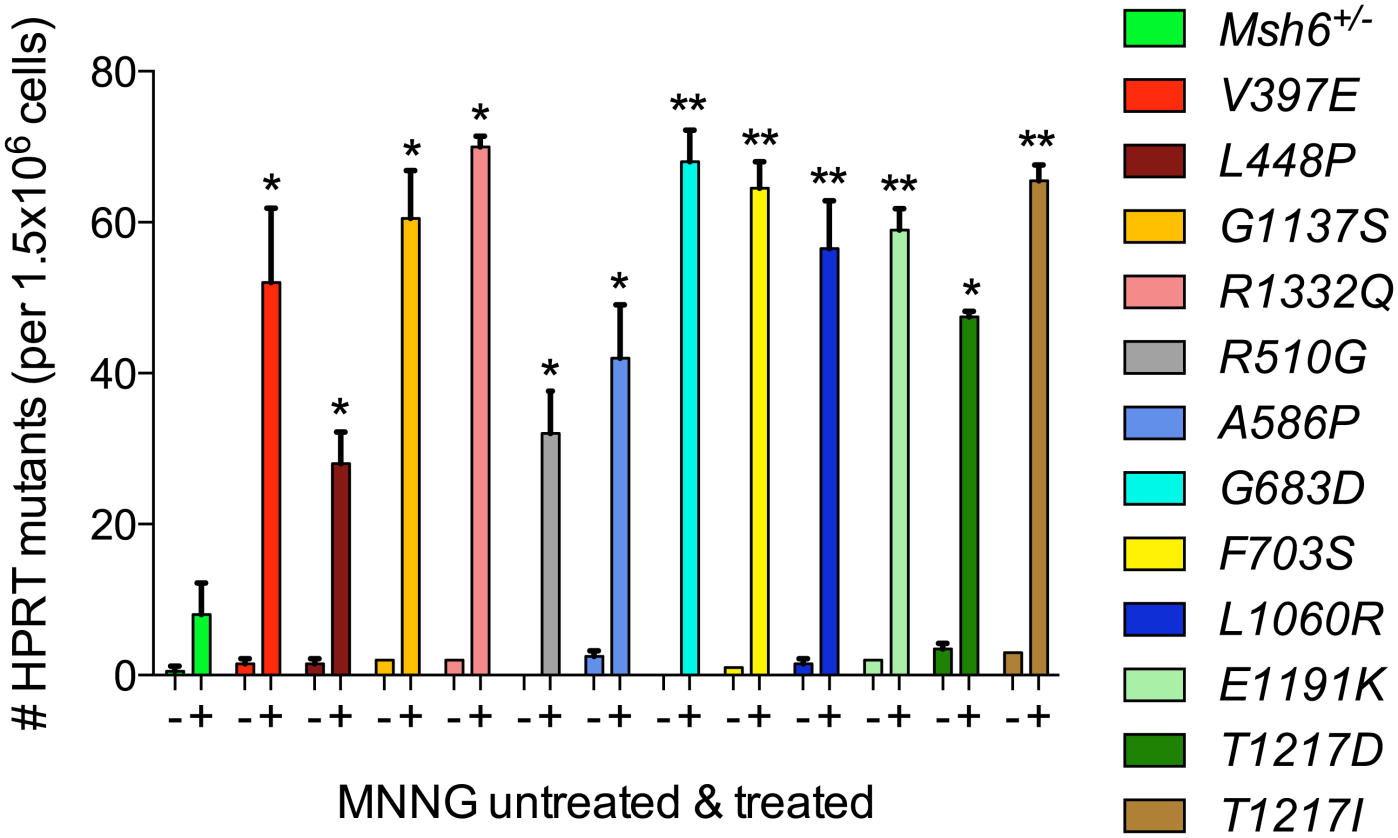
MNNG-induced mutagenesis in mESCs expressing *Msh6* variants. Variant MSH6 expressing mESCs were exposed to MNNG and the number of cells that consequently acquired mutations in *Hprt* quantified [43]. *Hprt*-defective mESCs were identified by long-term exposure to a high dose of 6TG (10 μg/ml). The spontaneous (-) and MNNG induced (+) mutation frequency was compared to MMR-proficient *Msh6*^*+/-*^ mESCs and MMR-deficient *Msh6* ^*V397E/-*^, *Msh6* ^*L448P/-*^, *Msh6* ^*G1137S/-*^, and *Msh6* ^*R1332Q/-*^ pathogenic controls. The statistical differences between MNNG-treated *Msh6*^*+/-*^ mESCs and MNNG-treated variant cell lines was calculated using a one-tailed, unpaired t-test with Welch’s correction. Asterisks indicate values significantly higher than those of the MNNG-treated MMR-proficient *Msh6*^*+/-*^ control: *P<0.05; **P<0.01.

In addition to increased spontaneous mutagenesis events, MMR-deficient cells also experience increased methylation-damage-induced mutagenesis [43]. To study the influence of the detected MMR attenuating *Msh6* variants on methylation-damage-induced mutagenesis, mESCs were exposed to the methylating DNA damaging agent N-methyl-N’-nitro-N-nitrosoguanidine (MNNG) and the number of cells that consequently attained mutations was quantified. In MMR-proficient cells, DNA replication across MNNG-induced *O*^6^-methylguanine lesions is impaired by futile cycles of MMR, ultimately leading to cell death and suppression of methylation-damage-induced mutagenesis. Under MMR-deficient conditions, however, the MNNG-induced mismatches are not recognized and remain in the genome leading to the accumulation of mutations. To provide a quick read out for the frequency of mutation accumulation, we measured the number of MNNG-exposed cells that became resistant to a high dose of 6TG for an extended period. Solely cells that carry an inactivating mutation in *Hprt* survive stringent 6TG treatment because HPRT is required for 6TG to behave as a DNA damaging agent. All detected *Msh6* variant cell lines showed an elevated MNNG-induced mutator phenotype when compared to the MMR-proficient *Msh6*^*+/-*^ mESCs (Fig 6).

### Phenotypic assessment of a non-detected *Msh6* variant

**Fig 7:**
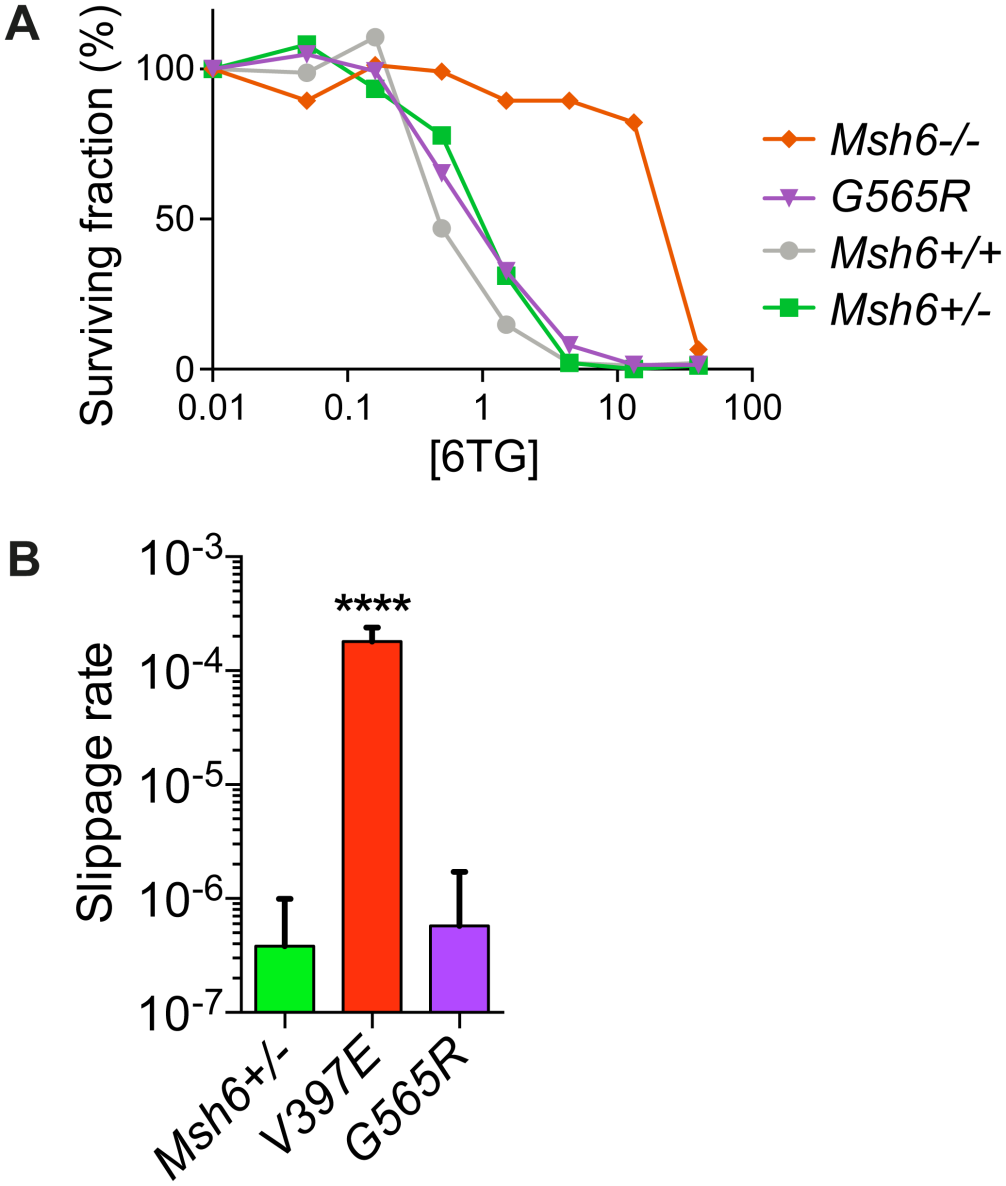
MMR capacity of *Msh6*^*G565R/-*^ mESCs. The MMR activity of *Msh6*^*G565R/-*^ mESCs was investigated using two assays. (A) 6TG survival assay. The colony-forming capacity of *Msh6*^*G565R/-*^ mESCs as well as MMR-deficient *Msh6*^*-/-*^ and MMR-proficient *Msh6*^*+/-*^ and *Msh6*^*+/+*^ cells was determined in response to increasing doses of 6TG. (B) MSI in the *Msh6*^*G565R/-*^ mESCs was investigated using the (G)_10_-*neo* slippage reporter. The slippage rate (the emergence of a Geneticin-resistant cell per cell division) in *Msh6*^*G565R/-*^ cells was compared to the rate in MMR-proficient *Msh6*^*+/-*^ and MMR-deficient *Msh6*^*V397E/-*^ control cell lines. Statistical differences were calculated using one tailed, unpaired t-test with Welch’s correction. **** indicates significantly higher than the mismatch repair proficient *Msh6*^*+/-*^ control: *P*<0.0001.

According to literature *MSH6-G566R* may be pathogenic [29,44], yet our screen did not identify this variant in 6TG-resistant colonies. Hence we investigated whether the MMR abrogating effect of *Msh6-G565R* could have been missed by the screen due to technical difficulties. Rather than applying 6TG selection after oligonucleotide-directed mutagenesis, we purified *Msh6*^*G565R/-*^ mESCs using a Q-PCR-based protocol [25] (S2C Fig) and subsequently examined their MMR capacity. Exposure of *Msh6*^*G565R/-*^ cells to increasing doses of 6TG revealed that they were equally sensitive to 6TG as *Msh6*^*+/-*^ cells (Fig 7A). In the MSI assay, *Msh6*^*G565R/-*^ mESCs did not experience significantly more slippage events than the MMR-proficient control (Fig 7B). Thus, *Msh6-G565R* did not attenuate MMR consistent with the oligonucleotide-directed mutagenesis screening result.

## Discussion

The results of our study demonstrate the oligonucleotide-directed mutagenesis screen we previously described for the characterization of *MSH2* VUS [22] can be extended to *MSH6* VUS. Combining oligo targeting in *Msh6*^*+/-*^ mESCs with 6TG selection and sequence analysis allows pathogenic *MSH6* variants to be distinguished from polymorphisms. The efficacy of the genetic screen was established in a proof of principle study with 4 known pathogenic *MSH6* mutations and 5 polymorphisms. This number was low because of the paucity of *MSH6* variants that were classified with 100% certainty. Not one of the 5 non-pathogenic variants was identified as MMR abrogating. Also, among the 26 *MSH6* VUS we subsequently analyzed, not one of the 4 variants classified as likely not pathogenic was identified as pathogenic by our screen. Finally, functional assays established that one of the VUS that was not detected as pathogenic by the screen indeed did not influence MMR activity (G565R). Hence the false positive rate of the screen, *i*.*e*., the chance the screen identified a VUS as MMR abrogating while it was *a priori* or *a posteriori* identified as (likely) non-pathogenic was <1/10, giving a specificity >90.0%. The sensitivity of the genetic screen is a measure of the false negative rate; it is the likelihood that a pathogenic mutation is not detected. All 6 InSiGHT classified pathogenic and likely pathogenic variants as well as the previously proven pathogenic G1139S mutation were recognized as MMR abrogating by the screen, translating to a sensitivity of >85.7%.

We used the oligonucleotide-directed mutagenesis screen to investigate the MMR capacity of 26 *MSH6* VUS. Eight of these were found in suspected-LS patients from two medical centers in the Netherlands. From this clinical cohort, the mouse equivalents of mutations R511G, A587P and F706S were detected by our screen and shown to abrogate MMR. However, R510G and F703S were detected in only 2/5 and 2/4 6TG-resistant colonies, respectively, that had retained two *Msh6* alleles, while the other pathogenic variants were present in virtually all colonies diploid for *Msh6* (Figs 2B, 3A and 3B). The poorer recovery of R510G and F7103S mutants may have been due to a lower success rate of LMO-mediated base-pair substitution. The pathogenic phenotype observed for these three variants is in line with clinical data: all three variants were detected in patients with MSI-H LS-related tumors and with a family history of LS-related tumors. In the case of VUS A587P and F706S, relatives with LS-related tumors carried the same mutation. IHC also demonstrated MSH6 was absent in the patients encoding MSH6-A587P and MSH6-F706S; the IHC data for MSH6-R511G were inconclusive.

The other 5 variants in the clinical cohort, A25S, E221D, G670R, R922Q and c.3438+6T>C, were not identified as MMR abrogating. VUS E221D, G670R and R922Q were found in patients who also harbored a second, known pathogenic mutation in one of the DNA MMR genes that was likely causative for the LS phenotype. E221D was also detected in a second patient who was 83 years old and did not have a family history suspicious for LS. MSH6-A25S was found in a typical LS tumor, *i*.*e*., a colon tumor showing MSI, loss of heterozygosity of *MSH6*, and loss of MSH6 protein expression. The patient however only had one relative with a colorectal tumor and this tumor was not MSI-high and stained positive for all MMR proteins. A previous *in vitro* study also suggested MSH6-A25S is not pathogenic [45]; it could be that the tumor arose due to a missed somatic mutation. VUS c.3438+6T>C was found in a patient with a family history suspicious of LS. We however do not know if the relatives with LS-associated cancers also carried this specific *MSH6* sequence variant. IHC failed in the index patient carrying the c.3438+6T>C variant, therefore we cannot exclude that a somatic mutation or *MLH1* hypermethylation caused the MSI in the tumor. Tumor tissue of one family member was tested and showed no MSI and normal IHC. It is also possible that the genetic screen was unable to identify c.3438+6T>C as pathogenic due to differences between the human and mouse *MSH6* sequences. While the *MSH6* coding sequence is highly conserved, intron sequences are more variable between species (S4 Fig shows human and mouse sequence around c.3438+6). Hence there is a chance that variant c.3438+6T>C affects splicing in man but not in mice. According to several splice site prediction programs (NNSPLICE, GeneSplicer, Human Splicing Finder), however, c.3438+6T>C does not affect splicing.

The other 18 *MSH6* VUS we studied were attained from literature and the InSiGHT database. The genetic screen found 5 of these variants abrogate MMR: G686D, L1063R, E1193K, T1219D and T1219I. The detection of G686D and L1063R is in line with their InSiGHT classification, which describes the mutations as likely pathogenic. Variant E1193K has previously been suggested to cause LS in studies that identified the mutation in patients with ECs that were MSI and did not stain for MSH6 [27,28]. Not much clinical data is available for VUS T1219D but *Msh6*^*T1217D*^ mice were demonstrated to have increased cancer susceptibility [46]. VUS T1219I has been described in a CRC patient who had a family history of CRC and a MSI tumor that stained positive for MSH6, the latter being consistent with the high levels of this variant protein we observed in mESCs. Both clinical and *in vitro* data indicate MSH6-T1219I abrogates MMR activity [37,45].

*MSH6* VUS R128L, R468H, V509A, Y556F, P623A, S666P, E983Q, R1095C, T1255M and R1304K were not identified as pathogenic in our screen. These sequence variants were classified as likely not pathogenic by InSiGHT, identified in patients with *MLH1* promoter methylation or with MSS and MSH6 positive tumors, or observed in patients for whom little clinical data was available. VUS S285I, G566R and T1142M were also not detected as MMR attenuating by our screen, yet they seem suspicious for pathogenicity based on available data. MSH6-T1142M was previously suggested to be probably pathogenic based on clinical data describing the variant in a 27 year old patient with polyps who met the Bethesda guidelines, had a 61 year old mother with polyps, and did not carry pathogenic mutations in any other MMR gene nor showed *MLH1* promoter methylation in the tumor [36]. VUS S285I and G566R were detected in CRC patients with MSI (low and high, respectively) tumors that had loss of heterozygosity of *MSH6* [29]. Cyr and Heinen [44] investigated the effect of these two mutations on mismatch binding and processing: variant S285I was not found to have a specific MMR attenuating effect but variant G566R was suggested to abrogate MMR by interfering with the ATP-dependent conformational change that must take place to activate downstream repair pathways upon mismatch binding. We therefore purified *Msh6*^*G565R/-*^ mESCs and assessed their MMR capacity. The *Msh6*^*G565R/-*^ cells behaved like MMR-proficient *Msh6*^*+/-*^ mESCs, confirming the result of the oligonucleotide-directed mutagenesis screen. Despite the good performance of our screen and the high amino acid conservation of MSH6, we cannot exclude *Msh6-G565R* was not identified as pathogenic due to differences between mice and men. To fully dissuade this argument we will need to develop the oligonucleotide-directed mutagenesis screen in human cells.

The oligonucleotide-directed mutagenesis screen presented here is a relatively simple tool that can be used to investigate the pathogenic phenotype of many *MSH6* VUS in parallel. While the evolutionary conservation of MMR justifies the use of mouse cells for the majority of VUS, testing of splice-site and intronic mutations necessitates adaptation to human cells. Also, as long as uncertainty exists about its specificity and sensitivity, functional testing needs to be combined with clinical data and *in silico* estimations to arrive at a reliable classification of VUS. Conforming the updated American College of Medical Genetics and Genomics (ACMG) standards and guidelines for sequence variant interpretation, we are currently transferring our functional tests to certified Clinical Genetics laboratories and creating an infrastructure where test results are compared and interpreted taking into account all available data. In this way, LS mutation carriers can be identified with the highest certainty and enrolled in tailored surveillance programs while relatives without the mutation can be excluded from surveillance.

## Materials and methods

### Oligonucleotide-directed mutagenesis screen to identify pathogenic *MSH6* variants

The genetic screen was developed in *Msh6*^*+/-*^ mESCs, which contain one active *Msh6* allele (*Msh6*^+^) and one *Msh6* allele that was disrupted by the insertion of a *puromycin* resistance marker (*Msh6*^-^) [24]. The *MSH6* variants under investigation were introduced into the *Msh6*^*+/-*^ mESCs by oligo targeting using LMOs [23]. 7x10^5^ *Msh6*^*+/-*^ mESCs were seeded in BRL-conditioned medium on gelatin-coated 6 wells and exposed to a mixture of 7.5 μl *Trans*IT-siQuest transfection agent (Mirus), 3 μg LMOs and 250 μl serum-free medium the following day. After 3 days, 1.5x10^6^ LMO-exposed cells were transferred to gelatin-coated 10 cm plates and subjected to 6TG (250 nM) (Sigma-Aldrich) selection. After 10 days the 18 largest 6TG-resistant colonies were picked. Cells that became 6TG-resistant due to loss of heterozygosity events were excluded from further analyses using a PCR specialized to detect the presence of both the disrupted and non-disrupted *Msh6* alleles [24]. 6TG-resistant mESCs that maintained both *Msh6* alleles were sequenced to confirm the presence of the planned mutation.

### Western blot analysis

Western blot analyses were performed as described in Wielders et al. [25]. Rabbit polyclonal antibodies against mMSH2 (1:500) [47] and mMSH6 (1:500) [24] as well as mouse polyclonal antibody against γ-Tubulin (1:1000; GTU-88 Sigma-Aldrich) were used as primary antibodies. Protein bands were visualized using IRDye 800CW goat anti-rabbit IgG and IRDye 800CW goat anti-mouse IgG secondary antibodies (Li-cor) and the Odyssey scan. The infrared fluorescent signals measured by the Odyssey scan are directly proportional to the amount of antigen on the Western blots, allowing quantification of the protein bands.

### Microsatellite instability assay

mESCs were electroporated with the (G)_10_-*neo Rosa26* targeting vector as described in Dekker et al. [48]. The (G)_10_-*neo Rosa26* targeting vector is composed of a promoterless *histidinol* resistance gene as well as a *neomycin* resistance gene (*neo*) that is rendered out of frame by a preceding (G)_10_-repeat [42]. Once electroporated, 10^6^ cells were seeded on gelatin-coated 10 cm plates in BRL-conditioned medium and exposed to Histidinol (3mM) (Sigma-Aldrich). Successful integration of the vector into the *Rosa26* locus of the Histidinol-resistant colonies routinely occurs at a frequency of ±95% and was confirmed by Southern blot analyses. The individual successfully targeted colonies were subsequently expanded to 10^7^ cells and transferred to gelatin-coated 10 cm plates at a density of 10^5^ cells per plate for Geneticin selection (600 μg/ml) (Life Technologies). After 10 days, the number of Geneticin-resistant colonies was counted and the slippage rate of the variant mESCs calculated using the formula: 0.6 x Geneticin^total^ = *N* x *p* x log (*N* x *p*), where Geneticin^total^ is the number of Geneticin-resistant colonies, *N* the number of cells to which the culture was expanded, and *p* the number of mutations per cell division. Experiments were performed in quadruplicate and statistical differences calculated using a one-tailed, unpaired t-test with Welch’s correction.

### MNNG-induced mutagenesis assay

The MNNG-induced mutagenesis assay was performed as described in Claij and te Riele [43]. 2.5x10^6^ variant mESCs were seeded on an irradiated mouse embryonic fibroblasts feeder layer in 10 cm plates and exposed to 0 or 4μM MNNG (Sigma-Aldrich) for 1h the following day. 40 μM *O*^6^-benzylguanine was present in the medium from 1h prior to the MNNG treatment until 6 days after, at which point 1.5x10^6^ cells were transferred to gelatin-coated 160 cm^2^ plates for 6TG selection (10 μg/ml). After two weeks of 6TG selection, the number of resistant colonies and hence the frequency of MNNG-induced *Hprt* mutants could be determined. Experiments were performed in duplo and the statistical difference between MNNG-treated *Msh6*^*+/-*^ mESCs and MNNG-treated variant cell lines calculated using a one-tailed, unpaired t-test with Welch’s correction.

### Generation of *Msh6*^*G565R/-*^ mESCs

*Msh6*^*G565R/-*^ mESCs were made as described by Wielders et al. [25]. Variant G565R was introduced into *Msh6*^*+/-*^ mESCs by oligo targeting and a pure *Msh6*^*G565R/-*^ mESC clone was obtained by consecutive rounds of seeding and mutation specific PCR: oligonucleotide-exposed cells were expanded and subsequently seeded on a 96-well plate at a density of 5000 cells per well. A mutation-specific quantitative PCR was used to identify wells that contained *Msh6*^*G565R/-*^ mESCs. Positive wells were reseeded at lower density and positive wells again identified by Q-PCR. A pure clone was finally obtained by seeding single cells per well. Sequence analysis confirmed the creation of *Msh6*^*G565R/-*^ mESCs.

### 6TG DNA damage response assay

The 6TG sensitivity of *Msh6*^*G565R/-*^ mESCs was investigated by exposing the variant cell line to increasing doses of 6TG, as described in Wielders et al. [49]. MMR-deficient *Msh6*^*-/-*^ and MMR-proficient *Msh6*^*+/-*^ and *Msh6*^*+/+*^ mESCs were taken along for comparison.

### Clinical data

We investigated the pathogenic phenotype of *MSH6* VUS that were found in suspected-LS patients at the Clinical Genetics departments of the Erasmus Medical Center Rotterdam and Radboud University Medical Center Nijmegen. We collected tumor characteristics, age at diagnosis, results of molecular diagnostics and germline mutation analysis, and family history from medical records. MSI analysis was performed with the Bethesda panel [50] or with the Promega pentaplex MSI analysis [51]. IHC for MLH1, MSH2, MSH6 and PMS2 protein was performed as described previously [52]. Germline mutation analysis of *MSH6* was performed by sequencing and multiplex ligation dependent probe amplification. The *in silico* prediction model PolyPhen [53] was used to estimate the chance of a variant being deleterious.

## Supporting information

S1 Fig Alignment of human and mouse MSH6 amino acid sequences demonstrating conservation of studied variants. Asterisks mark amino acids that are not conserved between the human (upper row) and mouse (lower row) MSH6 proteins. The positions of the studied MSH6 variants are highlighted: known pathogenic variants in red, known not-pathogenic variants in green, detected 6TG-resistant variants in mustard, non-detected variants in blue.
(PDF)

S2 Fig Sequences of *Msh6* variants detected by genetic screen and *Msh6*^*G565R/-*^ mESCs. *Msh6* sequences in mESCs expressing (A) pathogenic variants in proof of principle study, (B) VUS detected in 6TG-resistant colonies, and (C) variant *Msh6-G565R*. Note that in most cases the sequences are a superposition of the variant allele and the normal sequence of the *Msh6*^-^ allele. One-letter amino acid codes are annotated below the nucleotide sequences. *Msh6* WT is the wild-type *Msh6* sequence.
(PDF)

S3 Fig Location of the studied mutations in the MSH6 protein. The MSH6 domains are displayed in different colors [39,40]. The studied mutations are annotated according to their amino acid number and change. The detected variants are depicted above the MSH6 domains: in orange are the 4 mutations in the proof of principle study, in purple are the 6TG-resistant VUS. Undetected variants are displayed below the MSH6 domains: in green are the non-pathogenic variants in the proof of principle study, in blue are the VUS that did not give rise to 6TG-resistance.
(PDF)

S4 Fig Alignment of human and mouse sequences around human *MSH6 c*.*3438+6T*. Depicted are the exon and intron sequences around position *c*.*3438+6* in human *MSH6* (upper) as well as the corresponding mouse sequence (lower). The amino acid codons are marked in blue and green and the corresponding amino acids are indicated above and below the sequences. *hMSH6 c*.*3438+6T* and *mMSH6 c*.*3432+6T* are highlighted in red.
(PDF)

S1 Table Clinical data available for 18 *MSH6* VUS that were selected for screening from literature and the InSiGHT database. For each of the 18 VUS we aimed to collect clinical data describing the type of tumors found in patients encoding these mutations. Where no data is presented, we did not find this information about the specific *MSH6* variant in the consulted literature. Cancer type and age of onset are noted: CRC, colorectal cancer; EC, endometrium cancer; LS related, Lynch syndrome related tumor. We annotated the MSI status of each tumor: MSS, microsatellite stable; MSI-L, microsatellite instable low; MSI-H, microsatellite instable high. Tumor IHC is also presented: +, protein is present; -, protein is absent in tumor. Also indicated is whether the index patients met the Bethesda, Amsterdam I, Amsterdam II guidelines or not any of the guidelines, as well as the patients’ family cancer history. The reference column presents the literature from which the clinical data was retrieved. The InSiGHT classification is shown for each tumor: 1, not pathogenic; 2, likely not pathogenic; 3, uncertain; 4, likely pathogenic; 5, pathogenic; NA, not available. PolyPhen scores were calculated on http://genetics.bwh.harvard.edu/pph2/. In the final column the results from our screen are presented.
(DOCX)

S2 Table Clinical data of 8 *MSH6* variants collected from medical centers in the Netherlands. Clinical data was collected by the Erasmus Medical Center Rotterdam and Radboud University Medical Center Nijmegen for the 8 variants in the clinical cohort. The table annotates the sex and age of the patients as well as the types of tumors they developed: CRC, colorectal cancer; GaC, gastric cancer; UtC, urologic cancer. Tumor pathology (MSI, IHC and other tumor analysis) data is indicated: MSS, microsatellite stable; MSI-L, microsatellite instable low; MSI-H, microsatellite instable high; IHC+, protein is present in the tumor; IHC-, protein is absent; LOH, loss of heterozygosity of *MSH6*. The ‘Other variants’ column describes any other MMR gene variant that was detected in the patients. Whether the index patients met the Revised Bethesda guidelines is displayed as well as the patients’ family cancer history. The InSiGHT classification is shown for each tumor: 3, uncertain; 4, likely pathogenic; NA, not available. PolyPhen scores were calculated on http://genetics.bwh.harvard.edu/pph2/. In the final column the results from our screen are presented.
(DOCX)
