# Supplementary material for: Suspected Lynch syndrome associated MSH6 variants: A functional assay to determine their pathogenicity
Source: PLoS Genet. 2017 May 22;13(5):e1006765. doi: 10.1371/journal.pgen.1006765 (PMC5460888; doi:10.1371/journal.pgen.1006765)
Supplement: S1 Fig — Asterisks mark amino acids that are not conserved between the human (upper row) and mouse (lower row) MSH6 proteins. The positions of the studied MSH6 variants are highlighted: known pathogenic variants in red, known not-pathogenic variants in green, detected 6TG-resistant variants in mustard, non-detected variants in blue. (PDF) [file pgen.1006765.s001.pdf]

```
Human:      1-MSRQSTLYSFFPKSPALSDANKASARASREGGRAAAAPGASPSPGGDAAWSEAGPGPRPLARSASPPKAKNLNGGLRRSVAPAA   PTSCDFSPGDLVWAKM
Mouse:      1-MSRQSTLYSFFPKSPALGDTKKAAAEASRQG   AAASGASASRGGDAAWSEAEPGSRSAAVSASSPEAKDLNGGLRRASSSAQAVPPSSCDFSPGDLVWAKM
                               * **  * *   * ***   *   *           *  * ** *   * *  *       **** **** *

Human:    101-EGYPWWPCLVYNHPFDGTFIREKGKSVRVHVQFFDDSPTRGWVSKRLLKPYTGSKSKEAQKGGHFYSAKPEILRAMQRADEALNKDKIKRLELAVCDEPS
Mouse:    101-EGYPWWPCLVYNHPFDGTFIRKKGKSVRVHVQFFDDSPTRGWVSKRMLKPYTGSKSKEAQKGGHFYSSKSEILRAMQRADEALSKDTAERLQLAVCDEPS
                                   *                        *                    * *             *  ***  *

Human:    201-EPEEEEEMEVGTTYVTDKSEEDNEIESEEEVQPKTQGSRRSSRQIKKRRVISDSESDIGGSDVEFKPDTKEEGSSDEISSGVGDSESEGLNSPVKVARKR
Mouse:    201-EPEEEEETEVHEAYLSDKSEEDNYNESEEEAQPSVQGPRRSSRQVKKRRVISDSESDIGGSDVEFKPDTKQEGSSDDASSGVGDSDSEDLGTFGKGAPKR
                     *  *** **       **     *  **  *      *                         *     **       *    **** * *

Human:    301-KRMVTGNGSLKRKSSRKETPSATKQATSISSETKNTLRAFSAPQNSESQAHVSGGGDDSSRPTVWYHETLEWLKEEKRRDEHRRRPDHPDFDASTLYVPE
Mouse:    301-KRAMVAQGGLRRKSLKKETGSA KRATPILSETKSTLSAFSAPQNSESQTHVSGGNDSSGPTVWYHETLEWLKPEKRRDEHRRRPDHPEFNPTTLYVPE
                ***** * *   **   *  * *  * *    *  *           *      *   *             *              * ***

Human:    401-DFLNSCTPGMRKWWQIKSQNFDLVICYKVGKFYELYHMDALIGVSELGLVFMKGNWAHSGFPEIAFGRYSDSLVQKGYKVARVEQTETPEMMEARCRKMA
Mouse:    400-EFLNSCTPGMRKWWQLKSQNFDLVIFYKVGKFYELYHMDAVIGVSELGLIFMKGNWAHSGFPEIAFGRFSDSLVQKGYKVARVEQTETPEMMEARCRKMA
              *                        *              *        *

Human:    501-HISKYDRVVRREICRIITKGTQTYSVLEGDPSENYSKYLLSLKEKEEDSSGHTRAYGVCFVDTSLGKFFIGQFSDDRHCSRFRTLVAHYPPVQVLFEKGN
Mouse:    500-HVSKFDRVVRREICRIITKGTQTYSVLDGDPSENYSRYLLSLKEKEEETSGHTRVYGVCFVDTSLGKFFIGQFSDDRHCSRFRTLVAHYPPVQILFEKGN
               *  *                      *        *          **     *                                      *

Human:    601-LSKETKTILKSSLSCSLQEGLIPGSQFWDASKTLRTLLEEEYFREKLSDGIGVMLPQVLKGMTSESDSIGLTPGEKSELALSALGGCVFYLKKCLIDQEL
Mouse:    600-LSTETKTVLKGSLSSCLQEGLIPGSQFWDATKTLRTLLEGGYFTG  NGDSSTVLPLVLKGMTSESDSVGLTPGEESELALSALGGIVFYLKKCLIDQEL
                *    *      **              *        **  ***********  *           *      *          *

Human:    701-LSMANFEEYIPLDSDTVSTTRSGAIFTKAYQRMVLDAVTLNNLEIFLNGTNGSTEGTLLERVDTCHTPFGKRLLKQWLCAPLCNHYAINDRLDAIEDLMV
Mouse:    698-LSMANFEEYFPLDSDTVSTVKPGAVFTKASQRMVLDAVTLNNLEIFLNGTNGSTEGTLLERLDTCHTPFGKRLLKQWLCAPLCSPSAISDRLDAVEDLMA
                       *          **  *    *                               *                     ***  *     *    *

Human:    801-VPDKISEVVELLKKLPDLERLLSKIHNVGSPLKSQNHPDSRAIMYEETTYSKKKIIDFLSALEGFKVMCKIIGIMEEVADGFKSKILKQVISLQTKNPEG
Mouse:    798-VPDKVTEVADLLKKLPDLERLLSKIHNVGSPLKSQNHPDSRAIMYEETTYSKKKIIDFLSALEGFKVMCKVSGLLEEVAGGFTSKTLKQVVTLQSKSPKG
                  **  **                                                            ** **    *  *  *    **  * * *

Human:    901-RFPDLTVELNRWDTAFDHEKARKTGLITPKAGFDSDYDQALADIRENEQSLLEYLEKQRNRIGCRTIVYWGIGRNRYQLEIPENFTTRNLPEEYELKSTK
Mouse:    898-RFPDLTAELQRWDTAFDHEKARKTGLITPKAGFDSDYDQALADIRENEQSLLEYLDKQRSRLGCKSIVYWGIGRNRYQLEIPENFATRNLPEEYELKSTK
                    *  *                                             *   * *  **                   *

Human:   1001-KGCKRYWTKTIEKKLANLINAEERRDVSLKDCMRRLFYNFDKNYKDWQSAVECIAVLDVLLCLANYSRGGDGPMCRPVILLP EDTPPFLELKGSRHPCIT
```

```
Mouse:     998-KGCKRYWTKTIEKKLANLINAEERRDTSLKDCMRRLFCNFDKNHKDWQSAVECIAVLDVLLCLANYSQGGDGPMCRPEIVLPGEDTHPFLEFKGSRHPCIT
                                         *          *     *                       *         * *  *   *    *

Human:    1101-KTFFGDDFIPNDILIGCEEEEQENGKAYCVLVTGPNMGGKSTLMRQAGLLAVMAQMGCYVPAEVCRLTPIDRVFTRLGASDRIMSGESTFFVELSETASI
Mouse:    1099-KTFFGDDFIPNDILIGCEEEAEEHGKAYCVLVTGPNMGGKSTLIRQAGLLAVMAQLGCYVPAEKCRLTPVDRVFTRLGASDRIMSGESTFFVELSETASI
                                   ** *                   *           *       *     *

Human:    1201-LMHATAHSLVLVDELGRGTATFDGTAIANAVVKELAETIKCRTLFSTHYHSLVEDYSQNVAVRLGHMACMVENECEDPSQETITFLYKFIKGACPKSYGF
Mouse:    1199-LRHATAHSLVLVDELGRGTATFDGTAIANAVVKELAETIKCRTLFSTHYHSLVEDYSKSVCVRLGHMACMVENECEDPSQETITFLYKFIKGACPKSYGF
                *                                                       ** *

Human:    1301-NAARLANLPEEVIQKGHRKAREFEKMNQSLRLFREVCLASERSTVDAEAVHKLLTLIKEL
Mouse:    1299-NAARLANLPEEVIQKGHRKAREFERMNQSLQLFREVCLATEKPTINGEAIHRLLALINGL
                                             *        * ** ***  * *  *  **
```
