# Supplementary figures and images for: Suspected Lynch syndrome associated MSH6 variants: A functional assay to determine their pathogenicity
Source: PLoS Genet. 2017 May 22;13(5):e1006765. doi: 10.1371/journal.pgen.1006765 (PMC5460888; doi:10.1371/journal.pgen.1006765)

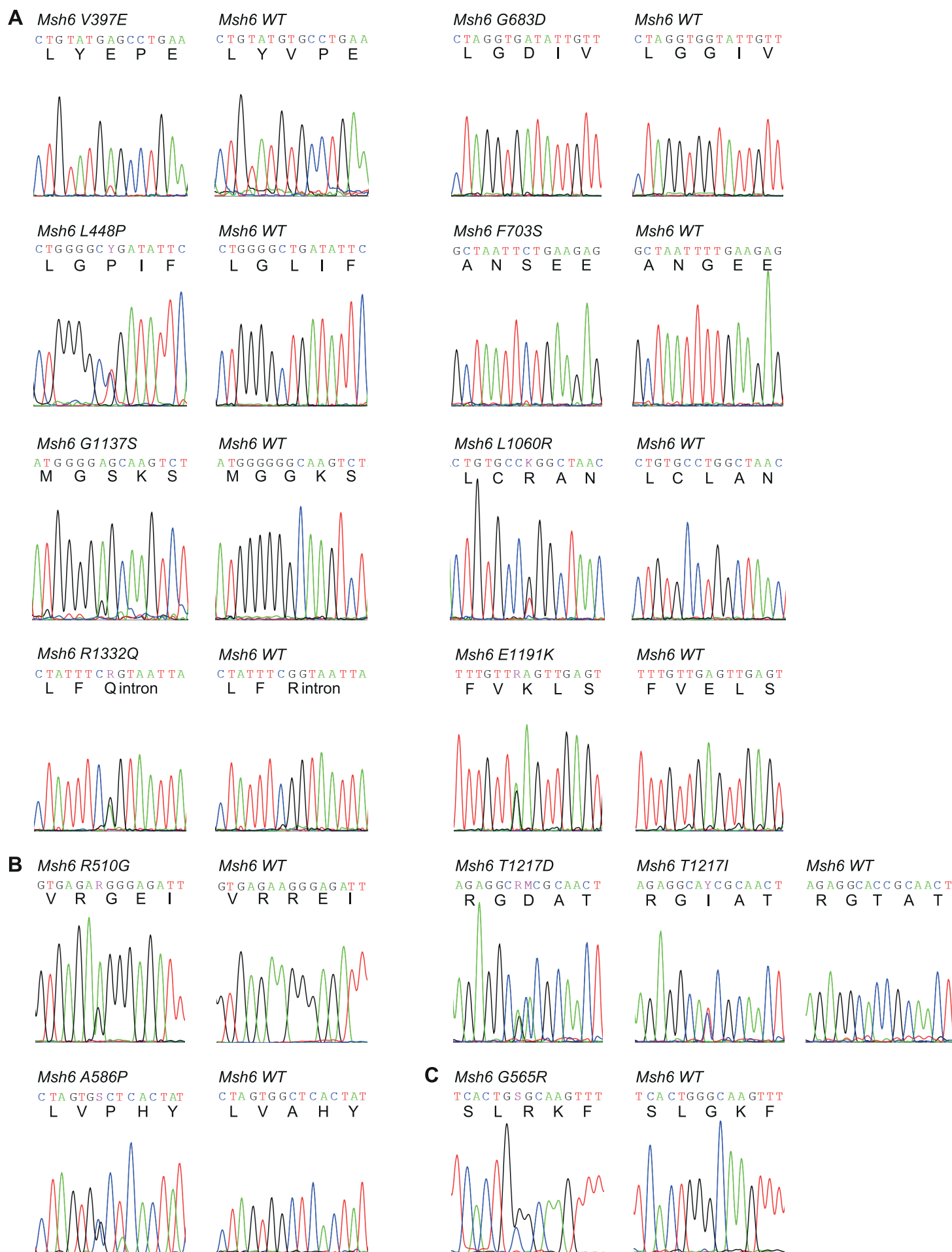

Supplement: S2 Fig — Msh6 sequences in mESCs expressing (A) pathogenic variants in proof of principle study, (B) VUS detected in 6TG-resistant colonies, and (C) variant Msh6-G565R. Note that in most cases the sequences are a superposition of the variant allele and the normal sequence of the Msh6- allele. One-letter amino acid codes are annotated below the nucleotide sequences. Msh6 WT is the wild-type Msh6 sequence. (PDF) [file pgen.1006765.s002.pdf]

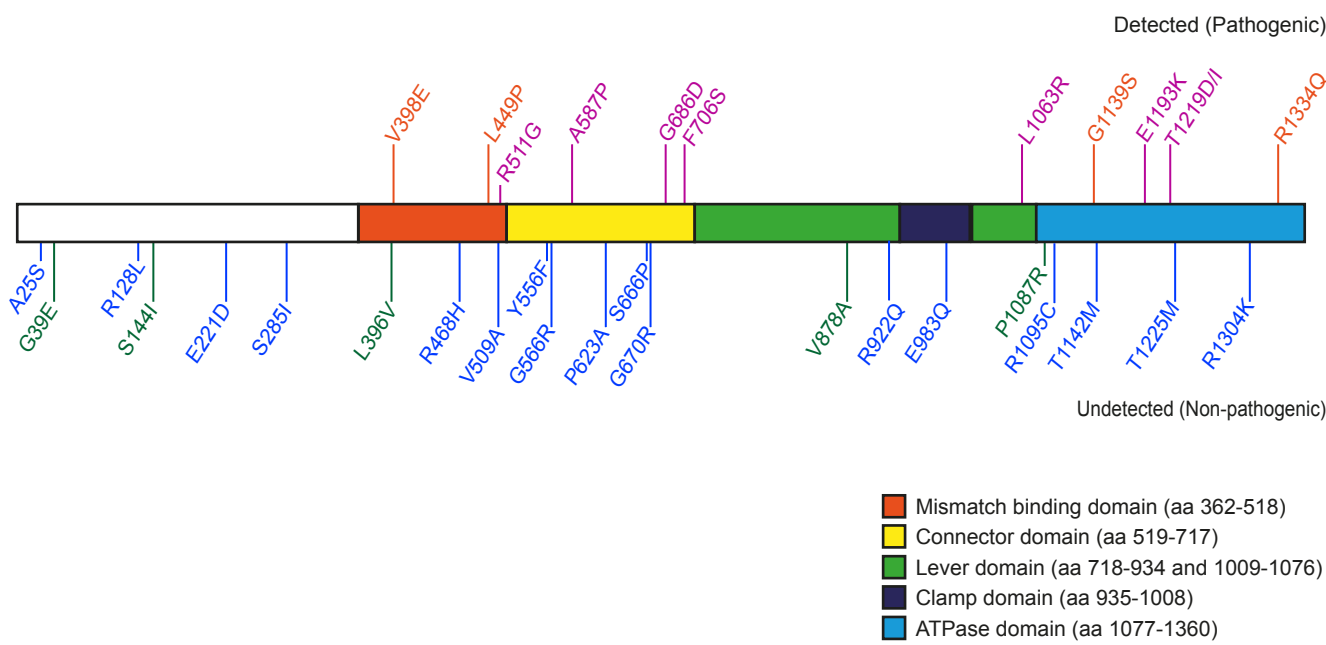

Supplement: S3 Fig — The MSH6 domains are displayed in different colors [39,40]. The studied mutations are annotated according to their amino acid number and change. The detected variants are depicted above the MSH6 domains: in orange are the 4 mutations in the proof of principle study, in purple are the 6TG-resistant VUS. Undetected variants are displayed below the MSH6 domains: in green are the non-pathogenic variants in the proof of principle study, in blue are the VUS that did not give rise to 6TG-resistance. (PDF) [file pgen.1006765.s003.pdf]
