# Supplementary material for: Suspected Lynch syndrome associated MSH6 variants: A functional assay to determine their pathogenicity
Source: PLoS Genet. 2017 May 22;13(5):e1006765. doi: 10.1371/journal.pgen.1006765 (PMC5460888; doi:10.1371/journal.pgen.1006765)
Supplement: S4 Fig — Depicted are the exon and intron sequences around position c.3438+6 in human MSH6 (upper) as well as the corresponding mouse sequence (lower). The amino acid codons are marked in blue and green and the corresponding amino acids are indicated above and below the sequences. hMSH6 c.3438+6T and mMSH6 c.3432+6T are highlighted in red. (PDF) [file pgen.1006765.s004.pdf]

```
Human amino acid sequence:   M  G  G  K  S  T  L  M  R  Q  INTRON
Human DNA sequence:         ATGGGGGGCAAGTCTACGCTTATGAGACAGGTAACTGATTCTTAA
Mouse DNA sequence:         ATGGGGGGCAAGTCTACACTCATAAGACAGGTAATTTGTTCTTCA
Mouse amino acid sequence:   M  G  G  K  S  T  L  I  R  Q  INTRON
```
