# Supplementary material for: Suspected Lynch syndrome associated MSH6 variants: A functional assay to determine their pathogenicity
Source: PLoS Genet. 2017 May 22;13(5):e1006765. doi: 10.1371/journal.pgen.1006765 (PMC5460888; doi:10.1371/journal.pgen.1006765)
Supplement: S1 Table — For each of the 18 VUS we aimed to collect clinical data describing the type of tumors found in patients encoding these mutations. Where no data is presented, we did not find this information about the specific MSH6 variant in the consulted literature. Cancer type and age of onset are noted: CRC, colorectal cancer; EC, endometrium cancer; LS related, Lynch syndrome related tumor. We annotated the MSI status of each tumor: MSS, microsatellite stable; MSI-L, microsatellite instable low; MSI-H, microsatellite instable high. Tumor IHC is also presented: +, protein is present; -, protein is absent in tumor. Also indicated is whether the index patients met the Bethesda, Amsterdam I, Amsterdam II guidelines or not any of the guidelines, as well as the patients’ family cancer history. The reference column presents the literature from which the clinical data was retrieved. The InSiGHT classification is shown for each tumor: 1, not pathogenic; 2, likely not pathogenic; 3, uncertain; 4, likely pathogenic; 5, pathogenic; NA, not available. PolyPhen scores were calculated on http://genetics.bwh.harvard.edu/pph2/. In the final column the results from our screen are presented. (DOCX) [file pgen.1006765.s005.docx]

**Table S1. Clinical data available for 18 *MSH6* VUS that were selected for screening from literature and the InSiGHT database.**

| **Variant** | **Tumor** | **MSI** | **IHC** | **Other tumor analysis** | **Amsterdam or Bethesda guidelines** | **Relatives with cancer** | **Reference** | **InSiGHT class** | **PolyPhen prediction** | **Screen result** |
| --- | --- | --- | --- | --- | --- | --- | --- | --- | --- | --- |
| R128L | EC 68 yrs | MSI-H | MSH2+ MSH6+  MLH1- | *MLH1* promoter methylation | No | •None | [27,28] | 3 | Possibly damaging | Not Pathogenic |
| S285I | CRC 69 yrs | MSI-L |  | Loss of *MSH6* heterozygosity in tumor |  | •Sister breast cancer 44 yrs and ovarian cancer 49 yrs  •Father lung cancer 69 yrs  •Mother CRC 74 yrs | [29] | 3 | Possibly damaging | Not Pathogenic |
| R468H | CRC <55 yrs | MSS | MSH2+ MSH6+  MLH1+ |  | No |  | [30] | 2 | Probably damaging | Not Pathogenic |
| R468H | CRC 50 yrs |  |  |  | Bethesda |  | [31] | 2 | Probably damaging | Not Pathogenic |
| V509A | CRC 45 yrs |  |  |  |  | •Father CRC  •Mother CRC | [32] | 2 | Probably damaging | Not Pathogenic |
| V509A | LS related |  |  |  |  |  | [33] | 2 | Probably damaging | Not Pathogenic |
| V509A | LS related |  |  |  |  |  | [34] | 2 | Probably damaging | Not Pathogenic |
| Y556F | EC 50 yrs |  |  |  | Amsterdam I & II | •Relative with same mutation developed CRC 50 yrs  •Three other relatives did not carry mutation but respectively developed CRC 43 yrs, CRC 42 yrs, Stomach cancer 29 yrs | [35] | 2 | Possibly damaging | Not Pathogenic |
| G566R | CRC 62 yrs | MSI-H |  | Loss of *MSH6* heterozygosity in tumor | No | •Sister CRC 73 yrs | [29] | 3 | Probably damaging | Not Pathogenic |
| P623A |  |  |  |  |  |  |  | 3 | Benign | Not Pathogenic |
| S666P | CRC 39 yrs |  |  |  | Bethesda |  | [31] | 3 | Possibly damaging | Not Pathogenic |
| G686D |  |  |  |  |  |  |  | 4 | Probably damaging | Pathogenic |
| E983Q |  |  |  |  |  |  |  | NA | Probably damaging | Not Pathogenic |
| L1063R | CRC 27 yrs |  |  |  |  | •Sibling CRC  •3 uncles/aunts CRC | InSiGHT database | 4 | Probably damaging | Pathogenic |
| R1095C |  |  |  |  |  |  |  | NA | Probably damaging | Not Pathogenic |
| T1142M | Polyps 27 yrs |  |  | No other pathogenic or unclassified variants. No *MLH1* promoter methylation | Bethesda | •Mother polyps 61 yrs | [36] | 3 | Probably damaging | Not Pathogenic |
| E1193K | EC 60 yrs | MSI-H | MSH2-  MSH6-  MLH1+ | No *MLH1* promoter methylations | No | •Brother skin cancer 60 yrs | [27,28] | 3 | Probably damaging | Pathogenic |
| E1193K | EC 59 yrs | MSI-H | MSH2+  MSH6 5%  MLH1+ | No *MLH1* promoter methylations | No | •None | [27,28] | 3 | Probably damaging | Pathogenic |
| T1219D |  |  |  |  |  |  |  | NA | Probably damaging | Pathogenic |
| T1219I | CRC 37 yrs | MSI-H | MSH2+  MSH6+  MLH1+ |  | Amsterdam II | •Parent CRC 36 yrs  •2^nd^ degree relative CRC  •2 3^rd^ degree relatives had cancer of unknown origins | [37] | 3 | Probably damaging | Pathogenic |
| T1225M | EC 61 yrs | MSI-H |  |  | No |  | [28] | 3 | Probably damaging | Not Pathogenic |
| T1225M | CRC 49 yrs | MSS | MSH6+ |  | No | •Parent CRC 49 yrs | [38] | 3 | Probably damaging | Not Pathogenic |
| T1225M | CRC 49 yrs |  |  |  | Amsterdam I & II | •Relative CRC 53 yrs  •Relative CRC 59 yrs | [35] | 3 | Probably damaging | Not Pathogenic |
| R1304K |  |  |  |  |  |  |  | 2 | Possibly damaging | Not Pathogenic |

For each of the 18 VUS we aimed to collect clinical data describing the type of tumors found in patients encoding these mutations. Where no data is presented, we did not find this information about the specific *MSH6* variant in the consulted literature. Cancer type and age of onset are noted: CRC, colorectal cancer; EC, endometrium cancer; LS related, Lynch syndrome related tumor. We annotated the MSI status of each tumor: MSS, microsatellite stable; MSI-L, microsatellite instable low; MSI-H, microsatellite instable high. Tumor IHC is also presented: +, protein is present; -, protein is absent in tumor. Also indicated is whether the index patients met the Bethesda, Amsterdam I, Amsterdam II guidelines or not any of the guidelines, as well as the patients’ family cancer history. The reference column presents the literature from which the clinical data was retrieved. The InSiGHT classification is shown for each tumor: 1, not pathogenic; 2, likely not pathogenic; 3, uncertain; 4, likely pathogenic; 5, pathogenic; NA, not available. PolyPhen scores were calculated on <http://genetics.bwh.harvard.edu/pph2/>. In the final column the results from our screen are presented.
