# Supplementary material for: Suspected Lynch syndrome associated MSH6 variants: A functional assay to determine their pathogenicity
Source: PLoS Genet. 2017 May 22;13(5):e1006765. doi: 10.1371/journal.pgen.1006765 (PMC5460888; doi:10.1371/journal.pgen.1006765)
Supplement: S2 Table — Clinical data was collected by the Erasmus Medical Center Rotterdam and Radboud University Medical Center Nijmegen for the 8 variants in the clinical cohort. The table annotates the sex and age of the patients as well as the types of tumors they developed: CRC, colorectal cancer; GaC, gastric cancer; UtC, urologic cancer. Tumor pathology (MSI, IHC and other tumor analysis) data is indicated: MSS, microsatellite stable; MSI-L, microsatellite instable low; MSI-H, microsatellite instable high; IHC+, protein is present in the tumor; IHC-, protein is absent; LOH, loss of heterozygosity of MSH6. The ‘Other variants’ column describes any other MMR gene variant that was detected in the patients. Whether the index patients met the Revised Bethesda guidelines is displayed as well as the patients’ family cancer history. The InSiGHT classification is shown for each tumor: 3, uncertain; 4, likely pathogenic; NA, not available. PolyPhen scores were calculated on http://genetics.bwh.harvard.edu/pph2/. In the final column the results from our screen are presented. (DOCX) [file pgen.1006765.s006.docx]

**Table S2. Clinical data of 8 *MSH6* variants collected from medical centers in the Netherlands.**

| **Variant** | **Tumor** | **MSI** | **IHC** | **Other tumor analysis** | **Other variants** | **Rev Bethesda guidelines** | **Relatives with cancer** | **InSiGHT class** | **PolyPhen prediction** | **Screen result** |
| --- | --- | --- | --- | --- | --- | --- | --- | --- | --- | --- |
| A25S | CRC 43 yrs female | MSI-H | MSH6- | LOH *MSH6* in tumor |  | Yes | •Father CRC 66 yrs (MSS, IHC normal) | 3 | Benign | Not Pathogenic |
| E221D | CRC or GaC 83 yrs female | No available tissue | No available tissue | No available tissue |  | No | •Daughter lung metastases, EC 33 yrs  •Father abdominal tumor 59 yrs | 3 | Benign | Not Pathogenic |
| E221D | CRC 44 yrs male | MSI-H | MSH2-MSH6- | Not analyzed | *MSH2* VUS L421P, which is pathogenic in our screen.  Cousin CRC 22 yrs only VUS L421P in *MSH2*, not *MSH6*. | Yes | •Mother renal cell carcinoma <48 yrs  •Uncle CRC 34 yrs  •Uncle bladder cancer 59 yrs  •Cousin CRC 22 yrs (MSI-H, IHC MSH2-MSH6-)  •Grandmother UtC <53 yrs | 3 | Benign | Not Pathogenic |
| R511G | UtC 62 yrs female | MSI-H | Suspect MSH2+ MSH6+ | Not analyzed |  | Yes | •Brother CRC 45 yrs (IHC normal) •Sister UtC 55 yrs | NA | Probably damaging | Pathogenic |
| A587P | CRC 59 yrs male | MSI-H | MSH6- | Not analyzed |  | No | •Sister UtC 56 yrs (MSI-H, IHC MSH6-, retention VUS in tumor)  •Grandfather CRC | NA | Probably damaging | Pathogenic |
| G670R | UtC 58 yrs CRC 71 yrs  female | MSI-H | MSH6- | Not analyzed | Pathogenic mutation (deletion exon 1+2) on same allele. | Yes |  | 3 | Probably damaging | Not Pathogenic |
| F706S | UtC 48 yrs female | MSI-H | Partly MSH6- | Not analyzed |  | Yes | •Sister CRC 36 yrs, ovarion cancer 38 yrs, breast cancer 50 yrs  •Father CRC 54 yrs (retention VUS in tumor)  •Paternal aunt CRC 62 yrs (One marker MSI and 2 markers MSS, IHC partly MSH2-MSH6-, retention VUS in tumor) | 4 | Probably damaging | Pathogenic |
| R922Q | CRC 39 yrs male | No available tissue | No available tissue | No available tissue | Pathogenic mutation *MSH6* (Asp936X) on other allele |  | •No other relatives with LS associated tumors | NA | Possibly damaging | Not Pathogenic |
| c.3438+6T>C | CRC 60 yrs female | MSI-H | Failed | Not analyzed |  |  | •Brother CRC 50 yrs  •Brother Pancreas carcinoma 72 yrs  •Sister CRC 38yrs  •Sister CRC 81 yrs •Nephew CRC 57 yrs (MSS, IHC normal) | 3 |  | Not Pathogenic |

Clinical data was collected by the Erasmus Medical Center Rotterdam and Radboud University Medical Center Nijmegen for the 8 variants in the clinical cohort. The table annotates the sex and age of the patients as well as the types of tumors they developed: CRC, colorectal cancer; GaC, gastric cancer; UtC, urologic cancer. Tumor pathology (MSI, IHC and other tumor analysis) data is indicated: MSS, microsatellite stable; MSI-L, microsatellite instable low; MSI-H, microsatellite instable high; IHC+, protein is present in the tumor; IHC-, protein is absent; LOH, loss of heterozygosity of *MSH6*. The ‘Other variants’ column describes any other MMR gene variant that was detected in the patients. Whether the index patients met the Revised Bethesda guidelines is displayed as well as the patients’ family cancer history. The InSiGHT classification is shown for each tumor: 3, uncertain; 4, likely pathogenic; NA, not available. PolyPhen scores were calculated on <http://genetics.bwh.harvard.edu/pph2/>. In the final column the results from our screen are presented.
